# Early Exposure to Traumatic Stressors Impairs Emotional Brain Circuitry

**DOI:** 10.1371/journal.pone.0075524

**Published:** 2013-09-20

**Authors:** Mayuresh S. Korgaonkar, Cassandra Antees, Leanne M. Williams, Justine M. Gatt, Richard A. Bryant, Ronald Cohen, Robert Paul, Ruth O’Hara, Stuart M. Grieve

**Affiliations:** 1 The Brain Dynamics Centre, University of Sydney Medical School - Westmead and Westmead Millennium Institute, Sydney, New South Wales, Australia; 2 Discipline of Psychiatry, University of Sydney Medical School: Western, Westmead Hospital, Sydney, New South Wales, Australia; 3 Department of Psychiatry and Behavioral Sciences, Stanford University, Stanford, California, United States of America; 4 School of Psychology, University of New South Wales, Sydney, New South Wales, Australia; 5 McKnight Institute on Aging, University of Florida, Gainesville, Florida, United States of America; 6 University of Missouri, St. Louis, Missouri, United States of America; 7 Sydney Medical School, University of Sydney and Royal Prince Alfred Hospital, Camperdown, Sydney, New South Wales, Australia; Beijing Normal University,Beijing, China

## Abstract

Exposure to early life trauma (ELT) is known to have a profound impact on mental development, leading to a higher risk for depression and anxiety. Our aim was to use multiple structural imaging methods to systematically investigate how traumatic stressors early in life impact the emotional brain circuits, typically found impaired with clinical diagnosis of depression and anxiety, across the lifespan in an otherwise healthy cohort. MRI data and self-reported histories of ELT from 352 healthy individuals screened for no psychiatric disorders were analyzed in this study. The volume and cortical thickness of the limbic and cingulate regions were assessed for all participants. A large subset of the cohort also had diffusion tensor imaging data, which was used to quantify white matter structural integrity of these regions. We found a significantly smaller amygdala volume and cortical thickness in the rostral anterior cingulate cortex associated with higher ELT exposure only for the adolescence group. White matter integrity of these regions was not affected. These findings demonstrate that exposure to early life trauma is associated with alterations in the gray matter of cingulate-limbic regions during adolescence in an otherwise healthy sample. These findings are interesting in the context that the affected regions are central neuroanatomical components in the psychopathology of depression, and adolescence is a peak period for risk and onset of the disorder.

## Introduction

Early life trauma (ELT) experienced during childhood and adolescence plays a critical role in the physical and mental development of an individual. Intensive exposure to stress during this critical developmental period can lead to chronic mental health conditions later in life [1]. Previous research has shown that exposure to traumatic stressors in early life has neurobiological implications (for a recent review see 2). The brain regions affected due to these adversities are the same found to be implicated in several psychiatric illnesses including depression and anxiety disorders [3]. These regions mainly include the emotional brain circuitry comprising of the anterior cingulate cortex [4] and the hippocampus-amygdala limbic regions [5,6]. An early impairment in these brain structures during brain development may affect the functioning of the hypothalamic-pituitary-adrenal axis later in life, altering cortical feedback-regulation and thus leading to increased vulnerability for stress-related psychiatric disorders [2]. Further, exposure to early life adversities moderate genetic risk for developing depression and anxiety [7,8]. There is also evidence that exposure to early life stressors may affect different brain regions at different phases during neuro-development. For example: childhood stress has been associated with reduced hippocampal volume in adults [9,10] but not in children [11]; whereas volume and white matter microstructure in the corpus callosum is found affected in both children [12] and older adults [12,13]. This may reflect age-specific interactions between early trauma exposure and brain structure.

Despite increasing knowledge about the gross morphometric and volumetric changes of these different brain regions following ELT exposure, little is known about the effect of ELT on cortical thickness. Grey matter measurements of volume are confounded by sulcal folding –cortical volume is a combination of thickness and surface area. Hence cortical thickness may be a more sensitive marker of grey matter alteration than is volume [14]. Recent work has also shown that both these neuroimaging measures maybe genetically independent [15]. Recent advances in automated methods for accurately measuring cortical thickness provide a powerful tool for studying this measure in large cohorts of subjects, increasing statistical power.

The current study is the first to determine whether exposure to ELT is associated with alterations in cortical thickness using these methods. Our focus on the cingulate and limbic emotion brain circuitry is based on evidence that this particular network is sensitive to stress exposure and is affected in numerous stress-related neuropsychiatric disorders [4-6]. We have previously demonstrated considerable heterogeneity in both cortical thickness-volume and white matter maturation in the limbic lobes [16], accordingly, in the current study we evaluate the individual sub regions of this brain system. We specifically hypothesize the anterior cingulate and the amygdala-hippocampus limbic regions to be altered with ELT exposure.

The second focus of this study was to use Diffusion Tensor Imaging (DTI) to determine whether any alterations in the macrostructural integrity of the emotion brain system were also evident at the microscopic level in the corresponding white matter. DTI is a non-invasive MRI technique that measures the integrity of white matter tracts *in vivo* by calculating the diffusion of water within specific regions of the brain. The metric commonly used is fractional anisotropy (FA), with lower FA values reflecting reduced white matter integrity. The decision to include DTI data is based on evidence that changes in cortical grey matter may be associated with changes taking place in the underlying white matter [16] and that white matter disruptions have been implicated in affective disorders [17]. There is also recent evidence that healthy participants with a greater familial risk for depression and exposure to more early life adversities have altered white matter integrity than those who had experienced low early life trauma exposure [18].

To test the impact of early trauma on neurobiological development, neuroimaging data from 352 participants across the entire lifespan, as part of the Brain Resource International Database (BRID) [19,20], was analyzed. We compared participants with high vs. low exposure to ELT. Additionally, this study extended previous research by investigating whether the effect of early trauma exposure on brain structure is reflected during a particular phase of the lifespan. We subdivided our sample into four age groups on the basis of major milestones in psychological adjustment (children, adolescents, young-middle adults or older adults). We hypothesized that individuals with High-Trauma exposure would exhibit reduced (a) cortical thickness, (b) volume in the anterior cingulate and amygdala-hippocampal limbic regions, and (c) microstructural integrity of the corresponding white matter pathways compared with those with Low-Trauma.

## Materials and Methods

### Ethics Statement

This study was performed in accordance with the ethical guidelines of the National Health and Medical Research Council of Australia approved by the Human Research Ethics Committee of Sydney West Area Health Services. Each participant provided written informed consent.

### Subjects

A total of 352 healthy individuals participated in a dual-site study as part of the BRID: Westmead Hospital (Sydney, Australia) (n= 160) and Wakefield Imaging (Adelaide, Australia) (n=192). Participants were community volunteers recruited via advertising over the web, community newspapers and flyers. Selection criteria included absence of a history of psychiatric diagnoses, of addiction to or heavy consumption of alcohol or other recreational drugs, and of any known neurological and/or learning disorders. Participants did not differ in general demographic distributions across sites, but did show differences in weight, body mass index (BMI) and years of education [16]. From those participants scanned at the Wakefield imaging site, 182 participants also had DTI data.

### Early Life Trauma Questionnaire

Exposure to ELT was assessed in a web-based battery, using the Brain Resource 19-item Early Life Stress Questionnaire. This questionnaire assesses the occurrence of specific early life stressors in the first 18 years of life, shown to have a psychological impact in childhood, including abuse, neglect, family conflict, illness/death, and natural disasters [13] (The complete questionnaire is provided as table S1). This scale has demonstrated reliability across cultures, and correlates with adult depression and anxiety syndromes [13,21]. We classified the current sample in terms of ‘High-Trauma’ exposure (participants with 3 or more early life stressors) versus ‘Low-Trauma’ exposure (0 to 2 stressors). This categorization for the same questionnaire has previously been used to evaluate effects of early life trauma exposure on the brain [7,22]. Demographic information for the cohort is provided in Table 1. The DTI subset showed a similar distribution of ‘low’ and ‘high’ ELT (Table 1).

**Table 1:** Demographic information for the ‘Low-Trauma’ and ‘High-Trauma’ exposure groups across the whole sample and each individual age group. Values in brackets are for the cohort with DTI data.

		Entire Sample	All Adults ≥18 yrs	Children (6-12yrs)	Adolescents (13-18yrs)	Young-Middle adults (19-50yrs)	Older adults (51-87yrs)
Low-Trauma	N	264 (141)	150 (59)	48 (37)	70 (48)	89 (32)	54 (24)
	No. of Males	137 (73)	74 (29)	25 (18)	39 (26)	39 (12)	32 (17)
	Mean ELT score	0.7 (0.8)	.79 (1.0)	0.4 (0.4)	0.7 (0.8)	0.8 (1.0)	0.8 (1.0)
High-Trauma	N	88 (41)	74 (33)	4 (4)	14 (6)	48 (17)	22 (14)
	No. of Males	35 (18)	24 (11)	4 (4)	7 (3)	16 (6)	8 (5)
	Mean ELT score	4.7 (1.7)	4.6 (5.0)	4.8 (4.8)	4.4 (4.2)	4.8 (5.4)	4.6 (4.9)

### Image acquisition

Magnetic Resonance Images were acquired using a 1.5 Tesla Siemens (Erlangen, Germany) Vision Plus system at Westmead Hospital, Sydney and a 1.5 Tesla Siemens Sonata at Perrett Imaging, Flinders University, Australia. 3D T1-weighted volumes were acquired in the sagittal plane using an MPRAGE sequence (TR=9.7 ms; TE=4 ms; Echo train: 7; Flip Angle=12°; TI=200 ms; NEX=1). A total of 180 contiguous 1 mm slices were acquired with a 256 x 256 matrix with an in plane resolution of 1 mm x 1 mm resulting in isotropic voxels. Diffusion tensor images were acquired using a DTI-EPI sequence (TR: 160 ms; TE: 88 ms; Fat Saturation; NEX: 4). A baseline image (b = 0) and 12 different diffusion directions were acquired with a b-value of 1250. 32 contiguous slices of 6.5 mm were acquired with an in-plane matrix of 128 x 128 at a resolution of 1.72 mm x 1.72 mm. Data from both sites has been previously cross-validated for comparison [20].

### Cortical thickness & volumetric analysis

**Figure 1:**
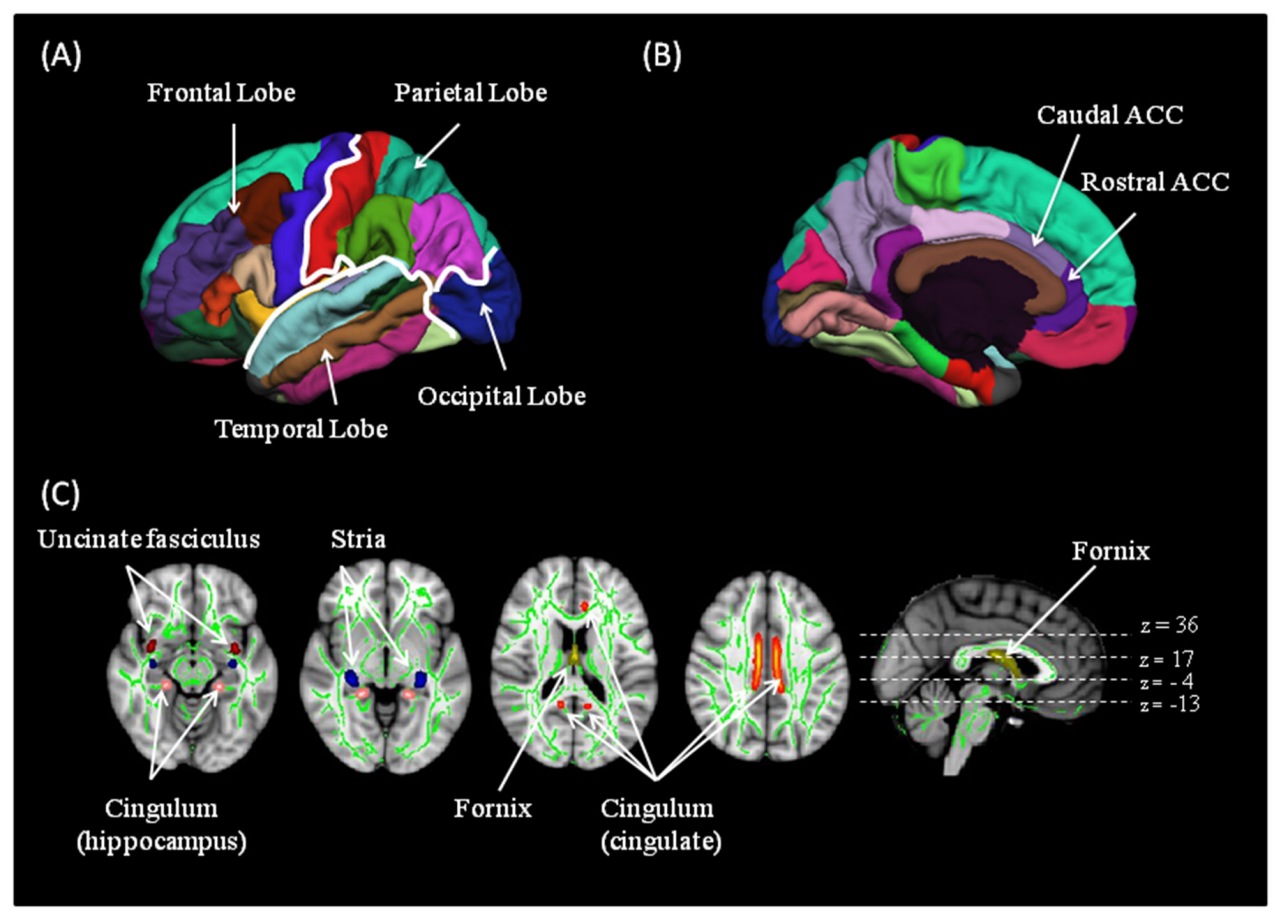
Grey matter and white matter regions of interest used in the study. (top row) Different brain lobes (A) and the rostral and caudal anterior cingulate sub-regions (B) parcelled using freesurfer for a representative dataset (bottom row (C)). Fractional anisotropy skeleton (in green) representing the major white matter tracts for all subjects is overlaid on the MNI standard brain. Sections of the tract sekeleton representing the cingulate and limbic white matter tracts are shown: cingulum cingulate bundle (orange); cingulum hippocampus bundle (pink); fornix (yellow); stria (blue) and uncinate fasciculus (red). Abbreviations: ACC, anterior cingulate cortex.

Cortical surface reconstruction and volumetric segmentation was performed in an automated manner on the 3D T1 weighted structural images using the Freesurfer image analysis suite (v4.3) (http://surfer.nmr.mgh.harvard.edu/). The technical details of these procedures are described elsewhere [16]. Briefly, for each subject, the boundary between the grey and white matter and the outer pial surface of the cortex were segmented. Cortical thickness measurements at each point across the cortical mantle were calculated as the closest distance from the gray/white boundary to the gray/CSF boundary [23]. The cortical surface and subcortical regions were automatically labeled [24]. For this study, the rostral and caudal anterior cingulate cortex (ACC) and amygdala and hippocampus limbic regions were analyzed. The individual cortical regions were also grouped into frontal, occipital, parietal and temporal lobes for lobe-wise analyses. Figure 1 shows the different brain lobes and the cingulate sub-regions parcelled using freesurfer for a representative dataset.

### DTI FA Data Analysis

DTI data were preprocessed and analyzed as described previously [17] using the TBSS (Tract Based Spatial Statistics) [25] software tools as part of the FMRIB Software Library (FSL) release 4.1.3 (http://www.fmrib.ox.ac.uk/fsl). The raw DTI data were corrected for head movement and eddy current distortions and diffusion tensor models fitted for all brain voxels. An FA image was generated for each participant.

These FA images were first aligned to the FMRIB58_FA template and affine transformed into MNI152 1mm^3^ standard space using the nonlinear registration tool FNIRT [26]. A white matter skeleton representing the centers of all white matter tracts common to all participants was generated from the average FA image for the cohort. This was thresholded to FA ≥ 0.2 to avoid peripheral tracts that are more vulnerable to intersubject variability and/or partial volume effects with grey matter. Each subject’s aligned FA image was then projected onto the mean FA skeleton by assigning each skeleton voxel by the maximum FA value found in a direction perpendicular to the tract. This accounts for any residual registration misalignments and variability in exact tract location between participants. We used the JHU ICBM-DTI-81 white matter labels atlas [27] to identify sections of the white matter skeleton corresponding to the cingulate and limbic white matter tracts (fornix, cingulum bundles near the cingulate and hippocampus, the crescent of the fornix/Stria Terminalis and the uncinate fasciculus) (see Figure 1C). An average FA value for each white matter tract was calculated.

### Statistical Analysis

Analyses of covariance (ANCOVA) procedures were used to contrast differences in cortical thickness and volume for the individual limbic and ACC sub-regions and FA for the associated white matter fiber tracts among the High-trauma and low-trauma group.

Using age groups instead of treating age as a continuous measures across the whole cohort was preferred because: 1. it allows to avoid bias of the ELT questionnaire (which records all trauma experienced till the age of 18years) in having inherently lower values for younger (less than 18years) participants in comparison to the older cohort because of their younger age; and 2. It allows determination of whether the changes in brain structure are specific to a particular period during the life span representing major milestones in psychological development. Hence analyses were done for the entire adult cohort (all participants ≥ 18years) and also separately for the individual age groups (i.e. children, adolescents, young and old adults). The children group had only four participants with high trauma exposure and any findings from this group were not considered suitable for interpretation. To explore if ELT exposure affects the other brain regions, these analyses were also done for the frontal, temporal, parietal and occipital brain lobes. Age and gender were controlled for in all analyses. Total brain volume was also controlled in all analyses using the volume data A threshold of p=0.05 (family wise error rate corrected using Holm-Bonferroni method) was used for statistical examinations.

## Results

### Cortical thickness

Cortical thickness was significantly reduced for the high trauma group in the rostral ACC (7.2%) only among adolescents (see table 2). No significant differences in cortical thickness due to exposure to ELT were present for the individual brain lobes.

**Table 2:** Means and statistical values for cortical thickness and volume for the cingulate and limbic regions as a function of early life trauma and age.

Brain region	Trauma group	All Adults (Ages ≥18)		Children (Ages 6-12)		Adolescents (Ages13-18)		Young Adults (Ages19-50)		Older Adults (Ages 51-87)	
		Mean (SD)	F & p-value	Mean (SD)	F & p-value	Mean (SD)	F & p-value	Mean (SD)	F & p-value	Mean (SD)	F & p-value
*Thickness (mm*)											
Rostral Anterior cingulate	Low-Trauma High-Trauma	2.843 (.208) 2.811 (.211)	.033. 856	3.080 (.244) 3.158 (.109)	.263. 610	3.027 (.211) 2.810 (.229)	11.20. 001*	2.886 (.193) 2.874 (.184)	.012. 912	2.756 (.209) 2.678 (.207)	2.40. 126
Caudal Anterior cingulate	Low-Trauma High-Trauma	2.538 (.176) 2.513 (.221)	.129. 720	2.807 (.207) 2.823 (.026)	.001. 976	2.684 (.165) 2.547 (.291)	5.16. 026	2.575 (.165) 2.575 (.195)	.090. 764	2.460 (.167) 2.411 (.168)	1.90. 172
*Volume (mm* ^*3*^)											
Rostral Anterior cingulate	Low-Trauma High-Trauma	4728 (816) 4727 (990)	.141. 708	5123 (793) 5985 (254)	2.267. 139	5006 (854) 4404 (721)	3.074. 083	4820 (808) 4795 (1106)	0.959. 329	4533 (774) 4673 (747)	.047. 829
Caudal Anterior cingulate	Low-Trauma High-Trauma	4480 (801) 4359 (958)	.482. 488	4943 (722) 5118 (357)	.021. 886	4906 (1050) 4706 (791)	.117. 734	4620 (817) 4375 (1063)	.745. 390	4166 (563) 4331 (800)	1.405. 240
Hippocampus	Low-Trauma High-Trauma	8276 (1030) 8201 (744)	2.692. 102	8395 (826) 8355 (501)	1.029. 316	8719 (908) 8419 (629)	.093. 761	8524 (978) 8245 (714)	1.463. 229	7852 (939) 8101 (855)	.236. 628
Amygdala	Low-Trauma High-Trauma	3055 (433) 3023 (363)	4.183. 042	3142 (343) 3429 (186)	1.634. 207	3263 (348) 3017 (358)	7.296. 008*	3106 (429) 3023 (351)	.057. 812	2951 (435) 3061 (414)	.348. 557

* Significant differences at family wise error corrected p < 0.05 (Holm-Bonferroni method)

### Volume

For the entire adult cohort, smaller volumes associated with high trauma exposure were found at a trend level (uncorrected p<0.05) for the amygdala (1.1% smaller, p=0.042 and ELT*age interaction: F=5.35, p=0.026). When stratified by the individual age cohorts, the high trauma group was found to have a significantly smaller amygdala volume only for adolescents (7.5%). Volume values for all cingulate and limbic sub-regions are summarized in Table 2. Volume for the brain lobes was not found to be significantly different due to exposure to ELT.

### FA measure for limbic-cingulate white matter tracts

There were no significant effects for FA for the limbic-cingulate white matter tracts due to exposure to ELT for either the entire adult cohort or for the individual age groups (See Table 3).

**Table 3:** Means and statistical values for Fractional Anisotropy for the cingulate & limbic white matter tracts as a function of early life trauma and age.

White matter tracts	Trauma group	All adults (Ages ≥18)		Children (Ages 6-12)		Adolescents (Ages 13-18)		Young Adults (Ages 19-50)		Older Adults (Ages 51-87)	
		Mean (SD)	F & p-value	Mean (SD)	F & p-value	Mean (SD)	F & p-value	Mean (SD)	F & p-value	Mean (SD)	F & p-value
Cingulum (Cingulate)	Low-Trauma High-Trauma	.411 (.041) .407 (.038)	.059. 808	.407 (.038) .390 (.042)	.834. 367	.419 (.028) .406 (.288)	1.519. 224	.426 (.039) .425 (.032)	.041. 841	.390 (.038) .382 (.034)	.020. 888
Cingulum (hippocampus)	Low-Trauma High-Trauma	.362 (.028) .368 (.035)	.372. 543	.327 (.030) .329 (.033)	.018. 894	.344 (.024) .346 (.036)	.131. 719	.369 (.024) .375 (.035)	.360. 551	.354 (.028) .359 (.036)	.644. 428
Fornix	Low-Trauma High-Trauma	.395 (.055) .385 (.046)	.022. 881	.406 (.029) .409 (.037)	.164. 688	.408 (.033) .395 (.044)	.594. 445	.423 (.040) .408 (.032)	1.899. 175	.356 (.054) .350 (.042)	.211. 649
Fornix (Stria)	Low-Trauma High-Trauma	.409 (.030) .412 (.037)	.670. 415	.400 (.025) .403 (.032)	.102. 752	.407 (.023) .397 (.027)	.982. 326	.421 (.026) .424 (.035)	.090. 765	.392 (.028) .395 (.036)	.211. 649
Uncinate Fasciculus	Low-Trauma High-Trauma	.475 (.036) .476 (.044)	.035. 852	.459 (.043) .473 (.095)	.613. 438	.489 (.029) .474 (.039)	1.646. 205	.478 (.039) .486 (.040)	.292. 592	.468 (.035) .462 (.048)	.035. 852

## Discussion

The present study utilized multiple structural MR imaging methods to examine the impact early life trauma has upon brain circuits associated with depression and anxiety. The main findings were concentrated during adolescence: cortical thickness of the rostral ACC and and the volume of the amygdala was reduced in the 13-18year high ELT exposure group only.

Neuroimaging techniques have allowed studying in-vivo brain changes with age. These are mainly developmental maturation changes until young adolescence, followed by an age-related slow decline in cortical volume and thickness during young-middle adulthood and an accelerated decline thereafter [28]. Particularly during childhood to young adulthood an early heterochronicity in grey matter maturation trajectories between individual sub-regions has been observed, with the timing and sequence of development shown to follow that of cognitive function [28]. At a cellular level, this variation in cortical maturation is likely to be due to a combination of myelination and synaptic pruning [29]. This period of neurodevelopment where the human brain is changing dynamically is also the key risk period for onset of mental disorders - half of all lifetime cases starting at the age of 14 years and almost three-fourths of those by 24 years [30]. Specifically, anxiety disorders have a median age-onset as early as 11years while depressive disorders at 30years. Using data from across the entire lifespan, we found that brain alterations due to exposure to ELT are apparent during this same neurodevelopmental phase. These findings are observed in an otherwise healthy sample, suggesting they may be trait-like markers for how trauma impacts the brain to confer susceptibility to depression and anxiety. However this warrants longitudinal follow-up and cannot be ascertained using cross-sectional data in this study.

While previous MRI studies on ELT exposure and brain structure have focused on volumetric measurements of specific brain regions, the current study is the first to evaluate alterations in cortical thickness. Cortical thickness is a measure of separation of the grey and white matter surfaces at every point of the cortex and more directly reflects change in grey matter in comparison to volume. Recent work has shown that both these measures are driven by distinct genetic factors [15,31], which emphasizes a complementary evaluation of both measures to identify suitable imaging phenotypes. This is particularly relevant for ELT, as exposure to trauma in early life has been shown to be a moderator of genetic risk in development of clinical depression [7]. Identifying imaging endophenotypes will help better understand how genetic risk and exposure to ELT translates to clinical symptomology.

Impacts on grey matter volume due to ELT were evident for the amygdala. This finding is noteworthy in the context of the known increased risk for depression and anxiety with ELT exposure. Previous research has demonstrated volume reductions in the hippocampus and the amygdala associated with ELT [11]. Our data showed robust evidence of decreased volume in amygdala but not in the hippocampus. Hippocampal findings in depression have shown to be highly variable across studies and mainly related to length and number of depressive episodes [32]. Negative life stressors have also shown to predict longitudinal hippocampal volume change [33]. Further, previous work using functional MRI have also shown that resting state connectivity of the posterior cingulate with the amygdala is altered in patients with PTSD related to ELT [34]. Amygdala dysfunction in adults with childhood maltreatment has also been previously reported [10]. Additionally there is clear evidence that negative life stressors have an immediate neurobiological impact on both these regions. A study on earthquake survivors observed both amygdala-hippocampal resting state functional connectivity and hippocampal volume to be reduced within 25 days post trauma in survivors [35,36].

Reductions in cortical thickness due to ELT exposure were in the rostral portion of the ACC. Our current findings extend on those reported in previous studies, which showed a volume reduction in the ACC among adults who had experienced childhood trauma [10,13]. For our analysis cohort, the volume of the ACC was not significant during adolescence (p=0.083) or for the adult cohort (p>0.05). We feel our current analysis represents a refinement on this previous observation, with the differences between these observations likely due to the different methodological approaches (volume analysis in VBM versus precise surface-based anatomical measurements in freesurfer). The ACC is a key structure, which regulates emotion reactivity via its direct connections to the amygdala, hence reductions in both these regions imply a significant structural alteration in the emotional reactivity circuitry that has these two regions as its core. Dysregulation of this same cingulate-limbic circuitry has often been observed in both depression and PTSD [37].

It is interesting to note, however, that significant differences in cortical thickness and volume were mainly found in the adolescent participants – reflecting vulnerability of these brain regions during this neurodevelopmental phase. Several other explanations are possible to explain these heterogeneous findings across age. Firstly, although these are cross-sectional data, it is possible that there is functional recovery of both these brain regions, after relief from early stressors, and the normalized volumes are a reflection of effective coping strategy in these participants to avoid clinical levels of depression and/or anxiety. It is also possible that our retrospective self-report measure underestimated ELT in the older adult cohort, who may have recall difficulties for more distal than current traumatic events. Thirdly, our cohort are free of a clinical history or diagnosis of depression or anxiety at time of participation, hence there may be a selection bias against structural damage due to ELT particularly in the older subjects – since subjects with neuropsychiatric disease presenting in later life which may be partly attributable to ELT would be excluded from the study. This latter point implies that the adolescents showing significant structural change in our study may represent a different population from the older subjects. However, only longitudinal data could further evaluate this interesting proposition. Despite these potential confounders, our data represent a fascinating “snapshot” of structural change due to exposed early trauma. The mostly adolescent loss in grey matter in these regions may represent a transient vulnerability to disorders associated with emotional dysregulation. Investigations of the interaction of genetic risk factors and ELT for neuropsychiatric outcomes observe a similar attenuation of these interactive effects in older adults [38].

The lack of significant findings with regard to the limbic and cingulate white matter fiber tracts is suggestive that the volume and cortical thickness loss may reflect a form of limited structural change due to ELT, where change was restricted to the cortex in healthy participants. Previous imaging studies in PTSD, major depression disorder patients, and healthy controls with greater familial risk for depression have reported altered FA values in the limbic white matter [17,18,39]. In our healthy cohort, the absence of clinically detectable symptoms may reflect the relative normality of the underlying white matter – even in the presence of detectable grey matter changes. Therefore, it’s possible that white matter connectivity effects may be detected in ELT cohorts with either a clinical diagnosis or with abnormal but non-clinically diagnostic scores.

The current study is limited by the fact that it does not take into account the different types of traumatic exposures or the timing of these exposures relative to neurodevelopment. There is some evidence supporting the concept that different brain regions may have their own developmental time windows when they are maximally vulnerable to the effects of ELT [40]. In addition to timing, it seems likely that certain types of traumatic events may have a greater impact on brain structure then others, although this can be challenging to evaluate given the frequency of multiple types of trauma exposure for individuals with any history of ELT. The absence of white matter alterations with ELT should be interpreted with caution especially in consideration of the DTI MRI sequence used in the study. Although this sequence was optimized for the available scanners at time of acquisition, the DTI spatial and directional resolution is limited in comparison to those available on newer scanners. This dataset however has been shown to be sensitive in mapping developmental changes across the entire lifespan [16]. The study is also limited by the small available sample size in the high ELT group for both adolescent and children. Closer look at the data verifies that outliers did not influence the main results seen for the adolescent group. However for the children group, the sample size is too low to draw any reliable interpretations.

In summary, the current analysis is the first to evaluate effects of early trauma on cortical thickness, volume and white matter associated with the emotional brain circuitry in the same cohort. Our findings show that exposure to early life trauma is associated with alterations in brain regions previously implicated in adult psychopathology, despite the absence of clinically significant psychiatric symptoms. These effects were most prominent during adolescence – a neurodevelopment period that is peak for risk and onset of depressive disorders. These structural markers will serve as useful endophenotypes in understanding how genetic, biological and environmental risk factors may have control in moderating effects of early life adversities in the development of depression and anxiety.

## Supporting Information

Table S1 **Early life stress questionnaire.**
(DOC)
