# Supplementary material for: Early Exposure to Traumatic Stressors Impairs Emotional Brain Circuitry
Source: PLoS One. 2013 Sep 20;8(9):e75524. doi: 10.1371/journal.pone.0075524 (PMC3779182; doi:10.1371/journal.pone.0075524)
Supplement: Table S1 — Early life stress questionnaire. (DOC) [file pone.0075524.s001.doc]

Supplementary table S1: Early life stress questionnaire

| 1. Were you born prematurely, or experience other birth complications? |
| --- |
| 1. Were you adopted? |
| 1. Did you undergo major surgery or repeated hospitalization? |
| 1. Did you experience a life-threatening illness or injury? |
| 1. Did you experience sustained bullying or rejection by schoolmates? |
| 1. Were you physically abused? |
| 1. Were you sexually abused? |
| 1. Were you emotionally abused? |
| 1. Did you experience extreme poverty or neglect? |
| 1. Did you witness first-hand a natural disaster such as earthquake, flood or fire? |
| 1. Was your house destroyed by fire or other means? |
| 1. Did you witness warfare? |
| 1. Did your parents’ divorce or separate? |
| 1. Were you separated for a long period from a parent, brother or sister? |
| 1. Was there sustained conflict within your family? |
| 1. Did one of your parents, a brother or sister die? |
| 1. Did one of your parents, a brother or sister experience a life-threatening illness? |
| 1. Did you witness domestic violence within your family? |
| 1. Did you witness or experience some other traumatic event? |
